# Effects of Immediate and Delayed Loading on the Outcomes of All-on-4 Treatment: A Prospective Study

**Published:** 2016-11

**Authors:** Hossein Najafi, Hakimeh Siadat, Solmaz Akbari, Amirreza Rokn

**Affiliations:** 1 Prosthodontist, Post Doc Student, Dental Implant Research Center, Dentistry Research Institute, Tehran University of Medical Sciences, Tehran, Iran; 2 Professor, Dental Implant Research Center, Dentistry Research Institute, Tehran University of Medical Sciences, Tehran, Iran; Department of Prosthodontics, Faculty of Dentistry, Tehran University of Medical Sciences, Tehran, Iran; 3 Assistant Professor, Dental Implant Research Center, Dentistry Research Institute, Tehran University of Medical Sciences, Tehran, Iran; Department of Periodontics, School of Dentistry, Tehran University of Medical Sciences, Tehran, Iran; 4 Professor, Dental Implant Research Center, Dentistry Research Institute, Tehran University of Medical Sciences, Tehran, Iran; Department of Periodontics, School of Dentistry, Tehran University of Medical Sciences, Tehran, Iran

**Keywords:** Dental Implants, Immediate Dental Implant Loading, Prospective Studies

## Abstract

**Objectives:**

The purpose of this study was to compare the outcomes of immediate and delayed rehabilitation of edentulous jaws by means of two straight and two tilted implants after one year of function.

**Materials and Methods:**

Thirty consecutive patients (16 males, 14 females) were enrolled in this study. Two anterior straight and two posterior tilted implants were placed in each patient. According to the implant insertion torque and the need for bone grafting, implants were loaded immediately (at 72 hours) or delayed (after four months) using a fixed metal resin prosthesis.

**Results:**

One axial implant failed in the delayed group after one year of loading, resulting in cumulative implant survival rate of 99.3%. The mean marginal bone loss was 0.84mm. No significant difference was found between axial and tilted implants in the two groups (P>0.05)

**Conclusions:**

Based on the results, immediate or delayed fabrication of final prosthesis on two tilted and two axial implants did not result in significant differences in survival rates or marginal bone loss.

## INTRODUCTION

Over time, rehabilitation of fully edentulous jaws by implant-supported prosthesis has gained popularity among patients and clinicians worldwide. However, insufficient bone volume due to long-term edentulism often complicates implant placement in the posterior region [1,2]. Different alternative treatments have been suggested to overcome anatomical limitations, including bone grafting techniques, short implants [3–5], inferior alveolar nerve transposition [6] and pterygomaxillary and zygomatic implants [7]. None of these therapeutic modalities have exhibited considerable surgical efficacy, with their own advantages, limitations and costs. The use of tilted implants has been proposed to avoid traumatization of the maxillary sinus or inferior alveolar nerve [8–14]. In 2003 and 2005, Malo et al, [15,16] presented clinical documentation of “All-on-4” concept in rehabilitation of edentulous mandible and maxilla. The “All-on-4” technique has been well-established in the clinical practice, and favorable survival rates and clinical results of immediate rehabilitation of fully edentulous jaws by two posterior tilted and two axial implants have been reported in the literature [15–19]. Immediate loading rehabilitation of edentulous jaws and its effectiveness has been extensively discussed in the literature [20–26]. Primary stability is suggested to be one of the main prerequisites for successful immediate loading techniques [27] and is influenced by bone quality, implant macro-design [28,29] and surgical techniques [16]. In standard “All-on-4” technique, patients receive an immediate provisional acrylic prosthesis a few days after surgery; but there are clinical situations, in which due to low insertion torque [27,30] or the necessity of bone augmentation simultaneous with implantation [26] immediate loading is not feasible. The purpose of this prospective study was to compare the clinical and radiographic outcomes of immediate and delayed restoration of edentulous jaws with the use of “All-on-4” treatment concept. The null hypothesis was that there would be no difference in survival rate and marginal bone level changes of delayed or immediately loaded implants and axial and tilted implants.

## MATERIALS AND METHODS

In this prospective study, patients with severely resorbed maxilla or mandible who required fixed prosthesis were consecutively enrolled and treated. This study was conducted in the Dental Implant Department of Faculty of Dentistry, Tehran University of Medical Sciences, Tehran, Iran. The study protocol was approved by the Ethics Committee of Tehran University of Medical Sciences (code no: 11149). All the patients signed informed consent forms.

### Selection criteria:

The inclusion criteria consisted of healthy individuals aged at least 18 years, atrophic fully edentulous mandible or maxilla, patients not willing to undergo bone augmentation procedures, and patient preference for fixed implant restoration. The exclusion criteria consisted of any systemic disease interfering with surgical intervention, severe para-functional habits, and poor cooperation in relation to follow-up visits. From April 2007 to January 2010, 30 consecutive patients fulfilled the study criteria and participated in the study.

### Surgical procedures:

After local anesthesia with 2% mepivacaine with 1:100,000 epinephrine (Scandinibsa; Inibsa Lab, Barcelona, Spain), a mid-crestal incision was made from the first molar to the first molar region. A mucoperiosteal flap was elevated and mental nerve foramen was located in the mandible. For localization of the anterior sinus wall, maxillary sinus prominence was followed. If necessary, a small window was opened to the sinus using a small bur. Malo Guide (Noble Biocare AB, Goteborg, Sweden) was used to guide implant positioning. A 2-mm hole was drilled in the middle of each jaw and the Malo Guide was inserted and adapted to the curvature of the alveolar ridge. All the patients received four implants (Branemark System MKIII or MKIV; Nobel Speedy Groovy; Nobel Replace select, Noble Biocare AB, Goteborg, Sweden). First, two distal implants were installed right at the mental foramina or anterior sinus wall with an inclination of 45° relative to the occlusal plane. Then, the axial implants were placed such that the most favorable implant distribution could be obtained, typically in the lateral incisor area. Considering the primary stability of the implants and the need for simultaneous bone augmentation, the patients were divided into two groups. If the insertion torque of all the four implants was ≥35 Ncm [20], the patient was placed in immediately loaded group (IL group). The multi-unit abutments were connected to the implants and an impression was taken. However, if the final torque of any implant was under 35 Ncm or if there was dehiscence or fenestration that required grafting at the time of implant placement, cover screws were connected and prosthetic fabrication was delayed (DL group). All the surgeries were performed by one surgeon (ARR).

### Prosthetic protocol:

An irreversible hydrocolloid (Alginoplast; Heraeus Kulzer GmbH & Co., Wehrheim, Germany) impression was made to obtain two casts from the residual ridge of all the patients before surgery. These casts were used to mold open custom trays and record base waxes [31,32].

In the IL group, 30° angled multi-unit abutment for posterior implants and straight multi-unit abutment for anterior implants were connected. All the straight abutments were torqued 30 Ncm and the angulated abutments were torqued 15 Ncm according to the manufacturer’s recommendation; these values were lower than the fixture insertion torque.

The square impression coping (Nobel Biocare AB) was adapted to the abutments. The impression copings were connected together with metal bars and autopolymerizing pattern resins using Duralay and GC Pattern Resin (Reliance Dental Manufacturing Co., IL, USA).

**Fig. 1:**
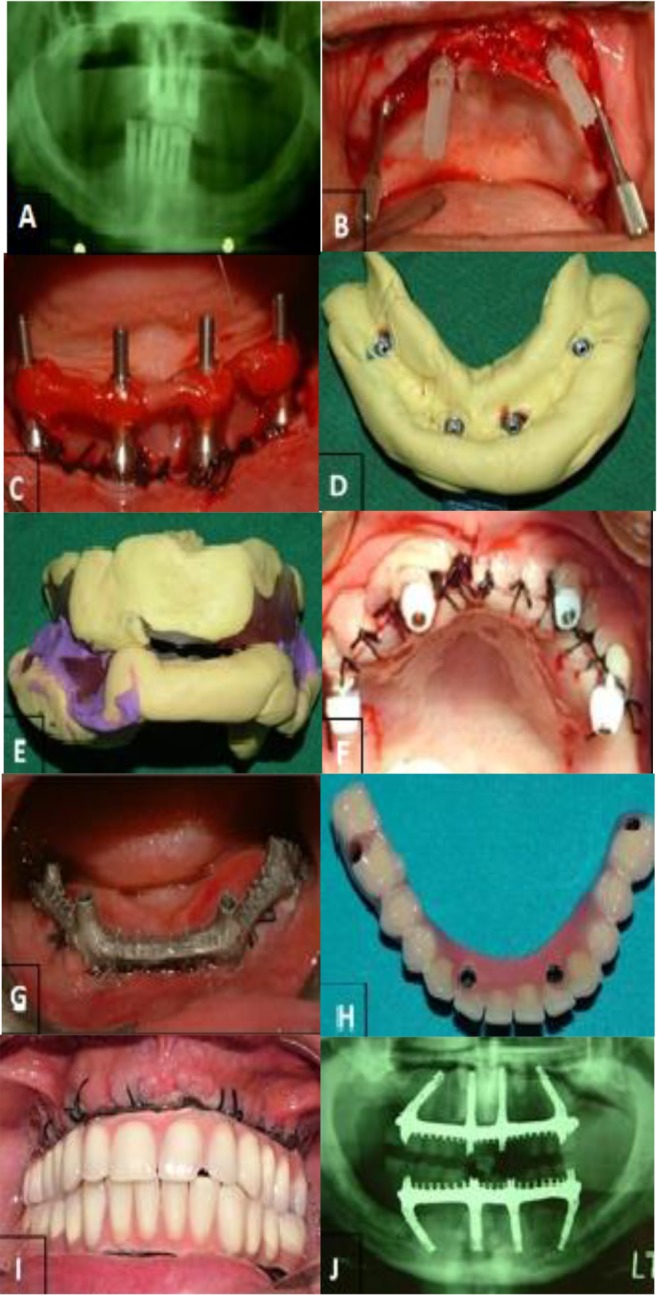
Images demonstrating (A) baseline X-ray (B) placing anterior and posterior multi-unit abutments, (C) fixation of the impression coping to metal bars and resin, (D) impression, (E) primary recording of occlusal vertical dimension and centric relation., (F) Placing healing caps and suturing, (G) checking metal bar in the mouth, (H) final prosthesis connection and (I) panoramic view obtained at delivery (J)

This would help achieve accurate molding. Addition silicone impression material (Elite HD + Regular Body; Zhermack, Kouigo, Italy) was used to take the final impression in all the patients. Record base wax rim was used to record the maxillary–mandibular relationship. Then, white healing caps were screwed on the multi-unit abutments and the flaps were sutured. The metal resin prosthesis was made in the same laboratory. On the third day after surgery, the final metal resin prosthesis was delivered (Fig. 1).

Occlusion was adjusted, and mutually protected occlusion was established. The patients were placed on soft diet [33].

Prosthetic procedures were carried out by one prosthodontist (H.N). Baseline radiographs were taken on the day of delivery. Since it is inconvenient for patients with severely resorbed mandible to hold intraoral films in the correct position, panoramic radiographs were taken for evaluation of marginal bone level. In the DL group, during the second surgery, which was carried out after four months, the abutments were connected and the remaining prosthetic procedures were the same as those in the IL group.

Oral hygiene measures were instructed. The patients were advised to use water irrigators as an adjunct to conventional measures. All the patients in the two groups were visited one week after prosthesis delivery for further occlusal adjustment and tissue healing evaluation. In absence of pain or any other complications, follow-up visits were scheduled at six and 12 months and yearly after that. Panoramic radiographs were taken in yearly visits.

### Data collection:

**Fig. 2:**
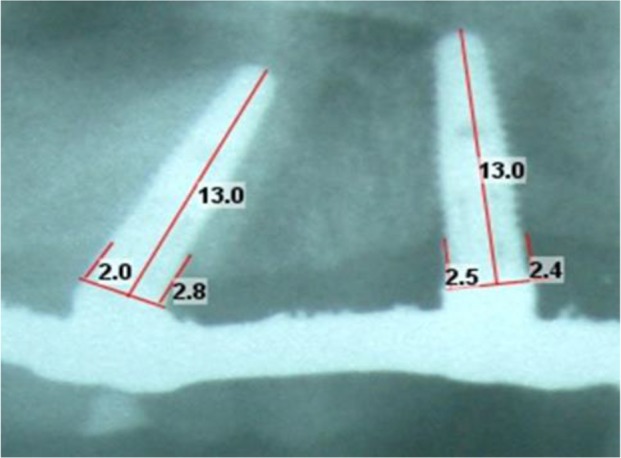
Radiograph was calibrated by the length of each implant. Radiographic bone loss was measured at the mesial and distal aspects of each implant

The following variables were evaluated:

- Implant survival: Implant should be stable, with no peri-implant radiolucency on radiographs and no suppuration or pain at the implant site.
- Marginal bone level: Radiographs were digitalized and imported to Romexis® version 2.6 software (Planmeca, IL, USA). The radiographs were calibrated by the length of each implant. Marginal bone loss was defined as the distance between the implant shoulder and the first bone-implant contact, at mesial and distal aspects of each implant in millimeters (Fig. 2). The mean value of mesial and distal bone loss represented bone loss of each implant.
- Prosthetic stability: If the prosthesis was in function without pain and mobility, it was considered stable. Abutment and prosthesis screw loosening or fractures and acrylic tooth chipping were recorded as complications.

### Statistical analysis:

For descriptive statistics, we reported mean±standard deviation for quantitative variables and frequency distribution for qualitative variables.

In addition, univariate tests such as the chi-square and independentsamples t-test were used to assess the relationship between different factors studied. Statistical significance was defined at P<0.05. SPSS version 16.0 (SPSS Inc., IL, USA) was used for statistical analyses.

## RESULTS

From April 2007 to January 2010, 30 patients (16 males and 14 females) with a mean age (± standard deviation) of 59.3±11.7 years (range 28–89 years) were treated and followed up for 32.5±13.6 months. No drop-out occurred. In general, 156 implants were placed in 39 jaws (25 mandibles, 14 maxillae). Thirteen cases (33.3%) were loaded in less than 72 hours (IL group) and 26 (66.6%) were rehabilitated four months after the surgery (DL group).

The opposing dentition was natural teeth, implant supported prosthesis or removable prosthesis. The distribution of the implant types and opposing dentition is shown in Table 1. After 13 months of loading, one maxillary straight implant in the DL group failed, which was replaced and the prosthesis was fabricated again. Therefore, the implant and prosthesis survival rates were 99% (104 implants) and 96.1% in the DL group, respectively. No implant or prosthetic failure occurred in immediately loaded group and the implant and prosthesis survival rate was 100%. Implant and prosthesis survival rate in the maxilla were 98.2% and 92.2%, respectively; while, both implant and prosthesis survival rates were 100% in the mandible.

**Table 1:** Distribution of implant types and opposing dentition

	** Opposing dentition **			** Implant type **		

	** Natural teeth **	** Implant-supported prosthesis **	** Removable prosthesis **	** Branemark MKIII, MKIV **	** Nobel Speedy Groovy **	** Nobel Replace Select **
** Number **	12 jaws	23 jaws	4 jaws	60	32	64
** Percentage **	30.7%	59%	10.3%	38.5%	20.5%	41%

### Mechanical complications:

The most common mechanical complication was acrylic tooth chipping (16 jaws, 41%).

Other complications were abutment screw loosening (one jaw, 2.5%), prosthetic screw loosening (two jaws, 5.1%) and prosthetic screw fracture (two jaws, 5.1%). The mean marginal bone loss in this study was 0.84±0.15mm (Table 2). No statistically significant difference was observed between the mean marginal bone loss of axial and tilted implants (P>0.05), maxillary and mandibular implants (P>0.05), or immediately loaded and delayed loaded groups (P>0.05).

**Table 2:** Marginal bone loss (in millimeters) according to implant location, angulation and loading mode

	** Jaws **		** Implant angulation **		** Type of loading **	

	** Mandible **	** Maxilla **	** Straight **	** Tilted **	** Immediate **	** Delayed **
** Marginal bone loss±standard deviation **	0.81±0.2	0.88±0.17	0.84±0.27	0.82±0.24	0.87±0.25	0.81±0.16

## DISCUSSION

The aim of the present study was to compare the outcomes of immediate and delayed rehabilitation of edentulous jaws with two tilted and two straight implants. The implant survival rates of 99.3% and 100% in delayed and immediate loaded groups, respectively, in the present study were in line with the results of other studies [11,15,16,19,34]. Beside the advantages of immediate loading such as shorter treatment time and high patient satisfaction, there is concern that immediate loading of dental implants may increase the risk of implant failure. The encouraging results of this study showed that by clinical consideration of each case and modification of prosthetic technique, similar results can be obtained with either immediate or delayed protocol. In the standard “All-on-4” technique by Malo et al, [15] patients receive an immediate provisional acrylic prosthesis and after six months the final prosthesis is fabricated; but in case of acrylic prosthesis fracture one or two implants might be overloaded and the probability of implant failure increases. The incidence rate of acrylic prosthesis fracture has been reported to be 11–27% in the literature [10 ,15,19,25]. However, in the present study the final prosthesis was fabricated with cast metal framework and delivered at the beginning; therefore, there was a decrease in the odds of such complications.

The chipping of the acrylic tooth was the most common mechanical complication (41%) in the present study. It was noted that most of the chippings took place in anterior region, where there are high shearing forces. All these chippings were restored chairside in dental office. The incidence of such complications was lower when the opposing dentition was removable denture. This finding is consistent with previous studies, which reported that the incidence of mechanical complication was higher when opposing dentition was implant-supported fixed prosthesis [12, 13, 35, 36]. After a mean of 32.5 months of follow-up, the mean marginal bone loss was 0.84mm, comparable with previous reports on “All-on-4” concept [15– 17,37]. Agliardi et al, [19] conducted a prospective study on 154 edentulous jaws rehabilitated immediately with fixed prostheses supported by four implants. Marginal bone loss after one year of loading was 0.9 and 1.2mm in the maxilla and mandible, respectively. Malo et al, [38] evaluated the long-term results of “All-on-4” treatment concept on 324 edentulous mandibles and reported marginal bone level of 1.81mm after five years of function. There was no significant difference in the mean marginal bone loss between straight and tilted implants in immediate and delayed loading groups and between maxillary versus mandibular implants. These outcomes were confirmed by some systematic reviews and meta-analyses [14,39–41]. In a systematic review by Del Fabbro and Ceresoli [41] it was observed that implant angulation (tilted versus straight), location (maxilla versus mandible), loading mode (immediate versus delayed) and restoration type (full versus partial prosthesis) had no significant influence on marginal bone level changes.

## CONCLUSION

Within the limitations of this study, successful outcomes suggested that immediate fabrication of final prosthesis on two tilted and two straight implants in edentulous jaws was safe and was not associated with higher marginal bone loss as compared to delayed loading protocol.
